# 3′-Daidzein Sulfonate Sodium Protects Against Chronic Cerebral Hypoperfusion-Mediated Cognitive Impairment and Hippocampal Damage via Activity-Regulated Cytoskeleton-Associated Protein Upregulation

**DOI:** 10.3389/fnins.2019.00104

**Published:** 2019-03-12

**Authors:** Qi Zeng, Zhihua Huang, Jiandong Zhang, Ruizhen Liu, Xiao Li, Jing Zeng, Hai Xiao

**Affiliations:** ^1^Department of Ultrasound, First Affiliated Hospital of Gannan Medical University, Ganzhou, China; ^2^School of Basic Medicine, Gannan Medical University, Ganzhou, China; ^3^Department of Pathology, First Affiliated Hospital of Gannan Medical University, Ganzhou, China; ^4^Key Laboratory of Prevention and Treatment of Cardiovascular and Cerebrovascular Diseases of Ministry of Education, Gannan Medical University, Ganzhou, China

**Keywords:** chronic cerebral hypoperfusion, learning and memory deficits, effective drug, 3′-daidzein sulfonate sodium, activity-regulated cytoskeleton-associated protein

## Abstract

The learning and memory impairment caused by chronic cerebral hypoperfusion (CCH) is permanent and seriously affects the daily life of patients and their families. The compound 3′-daidzein sulfonate sodium (DSS) protects against CCH-mediated memory impairment and hippocampal damage in a rat model. In the present study, we further investigated the underlying mechanisms of this effect in the rat two-vessel occlusion (2VO) and the oxygen and glucose deprivation (OGD) primary hippocampal neuron models. The hippocampal expression of the activity-regulated cytoskeleton associated protein (Arc) following DSS administration was detected *in vivo* and *in vitro* and behavioral testing was used to investigate the role of Arc in the DSS-mediated rescue of CCH-induced neurotoxicity. DSS increased hippocampal Arc expression both *in vivo* and *in vitro*. Arc overexpression increased and Arc knockdown decreased hippocampal neuronal densities in rat 2VO model, when compared to DSS treatment alone. Arc overexpression decreased and Arc knockdown increased apoptotic hippocampal neurons in rat 2VO and OGD primary hippocampal neuron models, when compared to DSS treatment alone. Arc overexpression enhanced and Arc knockdown inhibited the beneficial effect of DSS on 2VO-induced cognitive impairment. DSS restored the neuronal OGD-mediated phosphorylation decrease in protein kinase alpha (PKA), extracellular signal-regulated protein kinases 1/2 (ERK1/2) and cAMP response element binding protein (CREB), *in vitro*. PKA and ERK1/2 inhibition blocked the DSS-mediated effects on neuronal apoptosis and OGD-induced Arc downregulation. In conclusion, DSS protects against CCH-mediated cognitive impairment and hippocampal damage via Arc upregulation, which is activated by the PKA/CREB and ERK/CREB signaling pathways. Our study further confirms the potential use of DSS as an effective treatment for CCH-associated diseases.

## Introduction

Chronic cerebral hypoperfusion (CCH) can lead to a sustained cerebral blood flow reduction between 25 and 50% for over 6 months. Thus, CCH plays an important role in the pathogenesis of various nervous system disorders, such as vascular dementia, Binswanger’s disease and Alzheimer’s disease, among others (Sekhon et al., 1994, 1997). CCH impairs neuronal and capillary structure and function, leading to memory loss, and cognitive deficits (Sekhon et al., 1994, 1998). The learning and memory impairment caused by CCH is permanent and seriously affects the daily life of both patients and their families. Thus, there is a critical need for effective drug screening that can restore CCH-induced learning and memory impairments.

3′-Daidzein sulfonate sodium (DSS) is a new synthetic water-soluble compound derived from daidzein. DSS exhibited diversified pharmacological activities, such as anti-arrhythmia (Zeng et al., 2006a), anti-oxidation (Zeng et al., 2009), and anti-hypoxia (Zeng et al., 2006b). Furtherly, DSS can protect against myocardial and cerebral I/R injury (Zeng et al., 2009; Zhong et al., 2011), and improve mitochondrial functions after cerebral ischemia/reperfusion injury (Yuan et al., 2017). In addition, DSS inhibits neuronal apoptosis induced by cerebral ischemia-reperfusion (Liu et al., 2017) and DSS treatment provides neuroprotection in a focal cerebral ischemia rat model (Li et al., 2018a). Based on these results we predicted that DSS might play a role in improving impairments of learning and memory caused by CCH. This hypothesis has been proved by our previous study. We demonstrated that DSS protects against memory impairment and hippocampal damage caused by permanent carotid artery occlusion in the two-vessel occlusion method (2VO) (Li et al., 2018b). This model is a widely used experimental tool for the investigation of CCH-mediated neuronal damage and cognitive impairment (Ni et al., 1995; Ohta et al., 1997; Farkas et al., 2004). In the present study, we further investigated the underlying mechanisms behind this DSS-induced neuroprotection.

The activity-regulated cytoskeleton associated protein (Arc) was discovered by Dietmar Kuhl and Paul Worley in 1995 (Link et al., 1995; Lyford et al., 1995), as an immediate early neuronal gene that is required in synaptic plasticity and thereby aids in information storage optimization in the nervous system (Bramham et al., 2010). Synaptic plasticity is closely associated with all the major brain functions, such as learning and memory, nervous system development, and repair after injury (Citri and Malenka, 2008; Encalada et al., 2008; Bhat et al., 2017; Mansvelder et al., 2018). In addition, Arc has been proved to be involved in various neurological disorders (Epstein and Finkbeiner, 2018). It has been reported that Arc is involved in zinc-induced neurodegeneration (Mizuno and Kawahara, 2013). We demonstrated that Arc protein expression was decreased in the brain of the 2VO rat model and DSS can increase Acr expression (Liu et al., 2015). As it remains unclear whether DSS protects against the CCH-mediated cognitive impairment and hippocampal damage via Arc upregulation, we hereby investigate this further in the rat 2VO and oxygen and glucose deprivation (OGD) hippocampal models. In addition, we examine whether DSS regulates neuronal Arc expression through the protein kinase alpha (PKA) or extracellular signal-regulated protein kinase (ERK) signaling pathway, as seen in rat pheochromocytoma (PC12) cells (Waltereit et al., 2001; Epstein and Finkbeiner, 2018).

## Materials and Methods

### Animals

Sprague-Dawley (SD) rats (8 weeks, 200–240 g) were purchased from the Gannan Medical University Laboratory Animal Center (Gannan, China). All SD rats were housed in a pathogen-free animal house, and allowed free access to food and water. All SD rats were allowed to adapt to laboratory conditions for 7 days prior to any experimental procedures. All experimental procedures were approved by the Institute of Animal Care and Use Committee of Gannan Medical University (Gannan, China).

### Recombinant Adenoviruses

The negative control adenovirus (Ad-NC), the recombinant adenovirus that expresses Arc mRNA (Ad-Arc), and the short hairpin Arc RNA (Ad-shArc) were constructed by GenePharma (Suzhou, China).

### Experimental Animal Models

The 2VO model was engineered as previously described (Ni et al., 1994; Li et al., 2018b). Sham-operated controls refer to the SD rats that received the same surgical operation without carotid artery ligation and occlusion. All SD rats in the 2VO group were divided into four groups: (1) the 2VO group (*n* = 10); 3 weeks after 2VO surgery rats were gavaged with saline solution for 5 weeks, (2) the 2VO+0.1 mg/kg DSS group (*n* = 10); 3 weeks after 2VO surgery rats were gavaged with 0.1 mg/kg DSS for 5 weeks, (3) the 2VO+0.2 mg/kg DSS group (*n* = 10); 3 weeks after 2VO surgery rats were gavaged with 0.2 mg/kg DSS for 5 weeks, and (4) the 2VO+0.4 mg/kg DSS group (*n* = 10); 3 weeks after 2VO surgery rats were gavaged with 0.4 mg/kg DSS for 5 weeks. To identify the role of Arc in regulating the effects of DSS on 2VO-induced injuries, Ad-NC, Ad-Arc, or Ad-shArc was injected into the hippocampus (coordinates: 4.16 mm posterior; 2.0 mm lateral to Bregma; depth, 3.0 mm below the pia) through glass micropipettes glued onto a Hamilton syringe (Paxinos and Watson, 2004). Seven days after the adenoviral injection, the 2VO surgery was performed and 3 weeks later rats were gavaged with 0.4 mg/kg DSS for 5 weeks.

### Morris Water Maze

The Morris water maze assay was carried out at 5 weeks after DSS treatment. The Morris water maze assay was conducted in a circular black tank (Institute of Materia Medica, Chinese Academy of Medical Sciences, Beijing, China) of 150 cm in diameter containing 40 cm of water (23–25°C). A circular platform (9 cm in diameter) was placed 2 cm beneath the water level. The swim paths of the rats were tracked, digitized, and stored for further behavioral analysis. The water maze was divided into four quadrants (I, II, III, and IV). The rats were given four trials per day (30 min inter-trial intervals) for four consecutive days during the spatial learning phase. During the learning phase, each animal was randomly placed in a different quadrant, with the exception of the quadrant where the platform was placed in each trial. The maximum trial length was 60 s. When a rat did not find the platform within 60 s, the latency time was calculated as 60 s. After the rats were taken out of the pool, they were dried with towels and returned to their cages. The platform was removed during the probe test.

### Brain Tissues Isolation

After the 5-week DSS treatment, all rats were anesthetized using 10% chloral hydrate (3.5 mL/kg) and were perfused through the left cardiac ventricle with saline (20 mL), followed by 4% paraformaldehyde in 0.1 M phosphate buffer pH 7.4 (20 mL). Then, the brains were removed, the appropriate brain tissues were frozen in liquid nitrogen and the remaining brain tissues were post-fixed in the same fixative solution at 4°C for 2 h. The remaining brain tissues were then cryopreserved in 30% sucrose in phosphate buffer at 4°C. The appropriate frozen brain tissues were used for western blot analysis. The fixed brain tissues were used for terminal deoxynucleotidyl transferase-mediated 2′-deoxyuridine 5′-triphosphate nick-end labeling (TUNEL) assay, and Nissl staining.

### Neuronal Isolation and *in vitro* Experimental Groups

Hippocampal neurons were isolated and cultured as described previously (Seibenhener and Wooten, 2012). Neurons in their third division (passage) were exclusively used in this study. To analyze the effects of DSS on Arc and the PKA/ERK/cAMP response element binding protein (CREB) signaling pathways in an OGD neuronal model, cells were divided into five groups: (1) blank cell group; cells were maintained at 37°C in a humidified 5% CO_2_ incubator for 24 h, (2) the OGD group; cells were cultured in a sugar-free and serum-free culture medium in 94% N_2_, 1% O_2_, and 5% CO_2_ at 37°C for 24 h, (3) the OGD+DSS group; cells were cultured in a sugar-free and serum-free culture medium containing 2.5, 5, or 10 μmol/mL DSS in 94% N_2_, 1% O_2_, and 5% CO_2_ at 37°C for 24 h. To identify the role of Arc in regulating the effect of DSS on OGD-induced injuries, neurons were infected with Ad-NC, Ad-Arc, or Ad-shArc. Two days after adenoviral infection, cells were cultured in a sugar-free and serum-free culture medium containing 10 μmol/mL DSS in 94% N_2_, 1% O_2_, and 5% CO_2_ at 37°C for 24 h. To identify the role of PKA/ERK/CREB signaling pathways and Arc in regulating the DSS-mediated effects on OGD-induced injuries, neurons were cultured in a sugar-free and serum-free culture medium containing 10 μmol/mL DSS and the PKA inhibitor, H-89 (or the ERK1/2 inhibitor PD98059) in 94% N_2_, 1% O_2_, and 5% CO_2_ at 37°C for 24 h.

### Western Blot

The total proteins of hippocampal tissues and primary neurons were isolated using radioimmunoprecipitation assay buffer (RIPA). The protein concentration of all tissue lysates was determined with a bicinchoninic acid assay (BCA) protein assay kit. Same amount of total protein was separated on 10% sodium dodecyl sulfate polyacrylamide gel electrophoresis (SDS-PAGE) and transferred to polyvinylidene difluoride (PVDF) membranes (Bio-Rad, Hercules, CA, Unite States). The membranes were blocked with 5% non-fat powdered milk in Tris-buffered saline containing 0.1% Tween 20 (TBST) for 1 h at room temperature (RT) and then incubated overnight at 4°C with the appropriate primary antibodies: anti-Arc, anti-PKA (phospho T197; p-PKA), anti-ERK1 + ERK2 (phospho T202 + T204; p-ERK1/2), and anti-CREB (phospho S133; p-CREB) (Abcam, Cambridge, MA, Unite States). After washing in TBST, the membrane was incubated for 1 h at RT with horseradish peroxidase (HRP)-conjugated goat anti-rabbit antibody, and protein bands were visualized using the Immun-Star^TM^ HRP Chemiluminescence Kit (Bio-Rad). Glyceraldehyde-3-phosphate dehydrogenase (GAPDH) was used as the internal loading control.

### Immunohistochemistry (IHC)

Following drying at 67°C for 2 h, slides were de-waxed in xylene, rehydrated using a series of alcohol dilutions, and immersed in 3% hydrogen peroxide methanol solution for 15 min to block endogenous peroxidase activity. The slides were then pretreated with antigen retrieval buffer (citrate buffer [pH 6.0]) at 100°C for 2 min and subsequently incubated with 10% normal goat serum at RT for 15 min to decrease non-specific reactivity. Next, the slides were incubated overnight with anti-Arc antibody (Abcam) at 4°C. The slides were then rinsed three times with 0.01 M phosphate-buffered saline (PBS, pH 7.4) for 10 min, and the primary antibodies were detected using a secondary antibody (Envision; Dako, Glostrup, Denmark) for 30 min at 37°C. Next, the slides were washed with PBS and were stained with 3,3′-diaminobenzidine. Finally, the slides were counterstained using Mayer’s hematoxylin, dehydrated, and mounted. Non-neoplastic brain tissues were used as controls. Arc expression in the CA1 hippocampal region was visualized at 400× magnification with an Olympus light microscope (Tokyo, Japan).

### TUNEL Assay

The TUNEL assay was carried out using the *in Situ* Cell Death Detection Kit (Roche Molecular Biochemicals, Mannheim, Germany) according to the manufacturer’s instructions. The chromogen diaminobenzidine tetrahydrochloride (DAB) was used to detect apoptotic cells in brain tissues. The CA1 regions from each rat were inspected for apoptotic nuclei (TUNEL positive) that were stained yellow brown using light microscopy (400×; Carl Zeiss, Jena, Germany). To detect apoptotic cells in primary cultured hippocampal neurons, the chromogen fluorescein isothiocyanate (FITC) was used. Apoptotic cells (green nuclei) were quantified by visualization of 10 random visual fields per section at 200× magnification with a fluorescent microscope (Leica, Darmstadt, Germany).

### Nissl Staining

All animals were deeply anesthetized with 10% chloral hydrate (3.5 mL/kg) and transcardially perfused with 4% paraformaldehyde (w/v) in PBS. The brains were removed and dipped into fresh 4% paraformaldehyde for an additional 48 h at RT for post-fixation. Then, the samples were embedded in paraffin and sectioned (4 μm). The sections were washed and stained with a 0.5% cresyl violet solution for 10 min. Then, the sections were washed again with distilled water, dehydrated in an ethanol series gradient (95%, 1 min; 95%, 30 s; 100%, 1 min; and 100%, 1 min), and subsequently soaked three times in xylene, for 5 min each time. Finally, the sections were mounted on coverslips and imaged with a light microscope (400×; Carl Zeiss, Jena, Germany).

### Statistical Analysis

Statistical analyses were performed with the statistical package for the social sciences (SPSS) version 19.0 (IBM SPSS, Armonk, NY, United States). Descriptive data were expressed as mean ± standard deviation (SD). Intergroup comparisons were conducted using one-way analysis of variance (ANOVA), followed by *post hoc* tests of least significant difference (LSD) for multiple pairwise comparisons. *p* < 0.05 indicated a statistically significant difference.

## Results

### DSS Increased Arc Expression in the Hippocampi of the 2VO Rat Model *in vivo*, as Well as in Hippocampal Neurons After OGD *in vitro*

**Figure 1:**
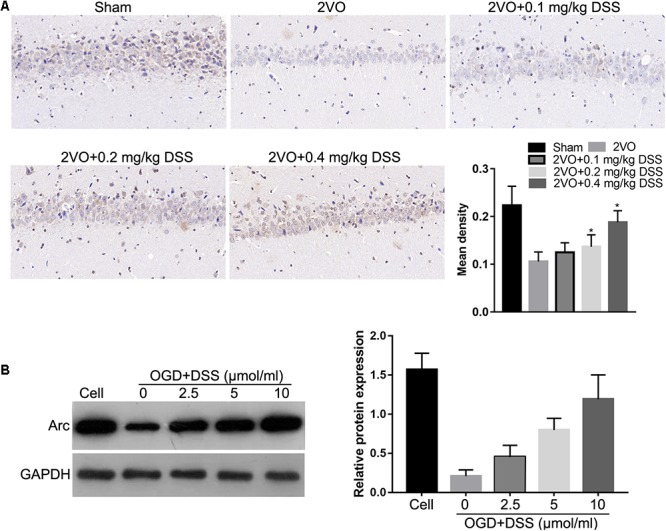
3′-Daidzein sulfonate sodium (DSS) increased Arc expression in the hippocampi of the 2VO rat model *in vivo* **(A)** and in primary hippocampal neurons following OGD, *in vitro* **(B)**. The bar graphs of **(A,B)** represent the mean density IHC quantification and the relative Arc expression normalized to GAPDH following western blotting, respectively. ^∗^*p* < 0.05, when compared to 2VO or OGD group.

To investigate the effects of DSS on Arc expression, IHC was used to detect Arc expression in the hippocampi of the sham, 2VO, 0.1 mg/kg DSS, 0.2 mg/kg DSS, 0.4 mg/kg DSS treated 2VO model groups. The results indicate that Arc expression was decreased in the 2VO group, when compared to the sham group, while DSS treatment restored Arc downregulation in a dose-dependent manner (Figure 1A). The effect of DSS on Arc expression was further evaluated in primary hippocampal neurons following OGD. Western blot analysis of Arc expression revealed a decrease in the OGD group, as compared to the cell group, while DSS treatment was able to restore this decrease in Arc expression in a dose-dependent manner (Figure 1B).

**Figure 2:**
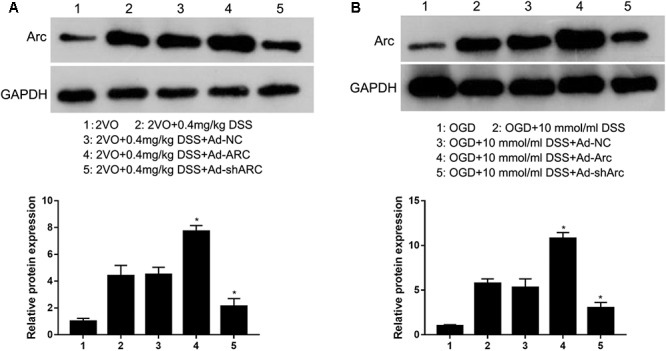
Arc protein was overexpressed or silenced in the hippocampus of the rat 2VO model **(A)** and in OGD primary hippocampal neurons treated with DSS **(B)** by infection with recombinant adenovirus that expressed either Arc mRNA (Ad-Arc) or a short hairpin Arc RNA (Ad-shArc). Bar graphs indicate the western blot quantifications of the relative Arc expression normalized to GAPDH. ^∗^*p* < 0.05, when compared to the negative adenovirus control (Ad-NC) group.

To identify the role of Arc in regulating the DSS-mediated effects on CCH-induced injuries, Ad-NC, Ad-Arc, or Ad-shArc was injected into the hippocampus of 2VO rats treated with 0.4 mg/kg DSS or used to infect OGD-cultured primary hippocampal neurons treated with DSS (10 μmol). Western blot analysis revealed that Arc expression increased -1.7-fold in the Ad-Arc group and decreased 52% in the Ad-shArc group compared to the Ad-NC group in the hippocampus of 2VO rats treated with 0.4 mg/kg DSS *in vivo* (Figure 2A). And Arc expression increased 2.1-fold in the Ad-Arc group and decreased 42% in the Ad-shArc group compared to the Ad-NC group in OGD-cultured primary hippocampal neurons treated with DSS (10 μmol) *in vitro* (Figure 2B). These results indicate that Arc was successfully overexpressed or silenced in both CCH models *in vivo* and *in vitro*, and both of these models can be used to examine the role of Arc in the regulation of DSS-mediated effects on CCH-induced injuries.

### Arc Overexpression Enhances and Arc Knockdown Inhibits the Neuroprotective Effects of DSS Exerted in the 2VO Rat Brains

**Figure 3:**
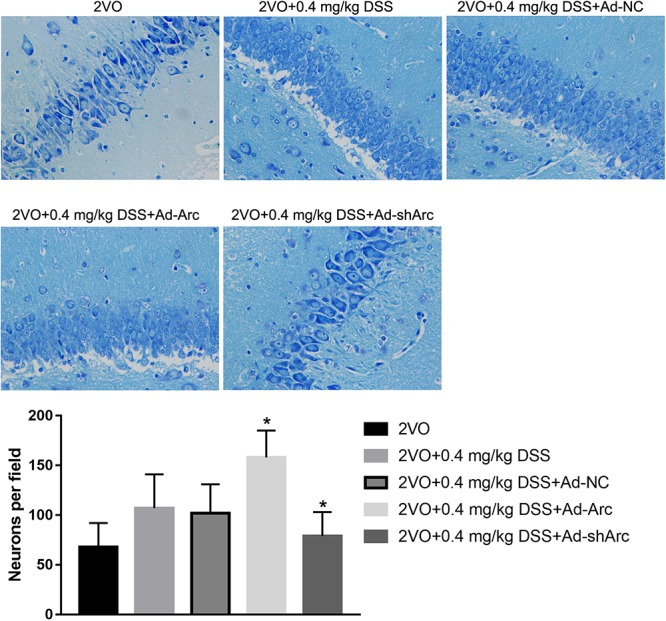
The effect of Arc overexpression or knockdown on hippocampal neuronal densities in 2VO rats treated with 0.4 mg/kg DSS. The top part of the figure shows the Nissl staining of the DG per animal group. The bottom part shows the neuronal density quantifications in the CA1 region per group. Bars represent mean ± SD for sample replications (*n* = 10). ^∗^*p* < 0.05, when compared to the 2VO+0.4 mg/kg DSS+Ad-NC group.

The role of Arc overexpression and Arc silencing on the protective DSS effect against neuronal damage observed in the 2VO rat model were examined by Nissl staining and TUNEL. Nissl staining revealed that neuronal densities in the CA1 hippocampal region of the 2VO+0.4 mg/kg DSS+Ad-Arc group were greater than those in the 2VO+0.4 mg/kg DSS+Ad-NC group (Figure 3). Moreover, neuronal densities in the CA1 hippocampal region of the 2VO+0.4 mg/kg DSS+Ad-shArc group were lower than those in the 2VO+0.4 mg/kg DSS+Ad-NC group (Figure 3). These results indicate that Arc overexpression increased and Arc knockdown decreased hippocampal neuronal densities in the 2VO model, when compared to DSS treatment alone.

**Figure 4:**
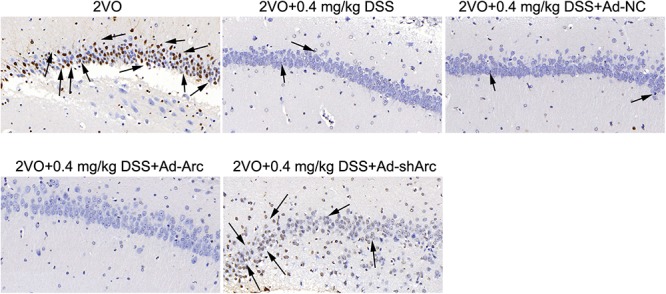
The effect of Arc overexpression and Arc knockdown on apoptotic neuronal number in the hippocampi of 2VO rats treated with 0.4 mg/kg DSS. Apoptotic neurons are indicated by black arrows.

The results of the TUNEL assay indicated that the number of apoptotic hippocampal neurons in the CA1 of the 2VO+0.4 mg/kg DSS+Ad-Arc group was smaller than the one seen in the 2VO+0.4 mg/kg DSS+Ad-NC group (Figure 4). The number of apoptotic hippocampal neurons in the CA1 of the 2VO+0.4 mg/kg DSS+Ad-shArc group was greater than that seen in the 2VO+0.4 mg/kg DSS+Ad-NC group (Figure 4). These results indicate that that Arc overexpression decreased and Arc knockdown increased apoptotic hippocampal neurons in the 2VO model, when compared to DSS treatment alone. All these results revealed Arc overexpression enhances the protective effect of DSS on 2VO-induced neuronal damage, while Arc knockdown inhibits this effect.

### Arc Overexpression Enhanced and Arc Knockdown Inhibited the Beneficial Effect of DSS on 2VO-Induced Cognitive Impairment

**Figure 5:**
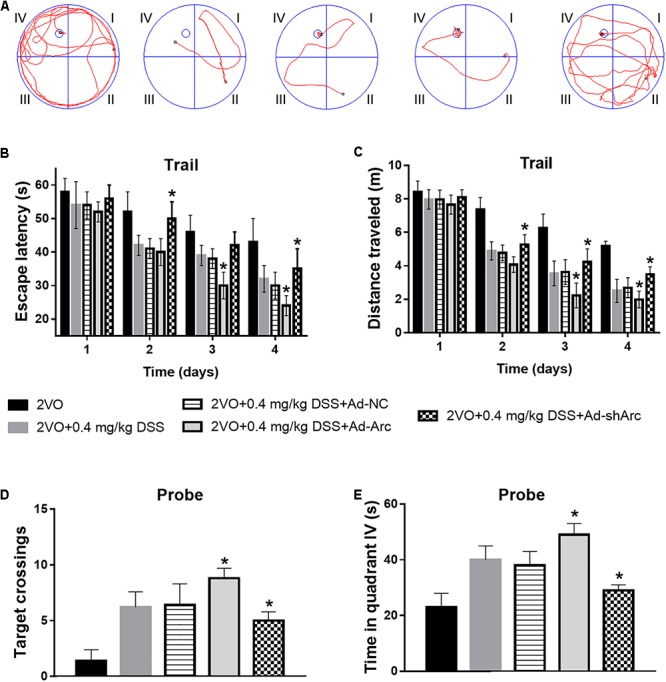
Effect of Arc overexpression and knockdown on the cognitive ability of 2VO rats treated with 0.4 mg/kg DSS. **(A)** Representative swim paths of each group at Day 4 during the place navigation test. **(B)** Mean daily escape latencies during the training phase are shown. **(C)** Mean distance traveled during the training phase are shown. **(D)** Summary of the number of crosses of the former platform area (swimming 120 s without platform). **(E)** Time spent in quadrant IV during the probe test. Bars represent mean ± SD for sample replications (*n* = 10). ^∗^*p* < 0.05, when compared to the 2VO+0.4 mg/kg DSS+Ad-NC group.

To investigate the effect of Arc overexpression and knockdown on the cognitive ability of 2VO rats treated with 0.4 mg/kg DSS, behavioral testing in the Morris water maze was carried out 5 weeks after DSS gavage. Representative swim paths of each group at Day 4 of the place navigation test are shown in Figure 5A. During the spatial learning phase, the rats in the 2VO+0.4 mg/kg DSS+Ad-Arc group exhibited shorter escape latencies and traveled distances than the rats in the 2VO+0.4 mg/kg DSS+Ad-NC group (Day 2 to Day 4 in the water maze task; Figure 5B,C). The rats in the 2VO+0.4 mg/kg DSS+Ad-shArc group exhibited longer escape latencies and traveled distances than the rats in the 2VO+0.4 mg/kg DSS+Ad-NC group (Day 2 to Day 4 in the water maze task; Figure 5B,C). During the probe trial, the number of times the rats crossed the platform area, as well as the time spent in quadrant IV for the 2VO+0.4 mg/kg DSS+Ad-Arc group was higher than the one observed in the 2VO+0.4 mg/kg DSS+Ad-NC group (Figure 5D,E). Also, the number of times the rats crossed the platform area, as well as the time spent in quadrant IV for the 2VO+0.4 mg/kg DSS+Ad-shArc group were smaller than that for the 2VO+0.4 mg/kg DSS+Ad-NC group (Figure 5D,E). These results indicate that Arc overexpression enhances and Arc knockdown inhibits the beneficial effect of DSS on 2VO-induced cognitive impairment.

### Arc Overexpression Enhanced and Arc Knockdown Inhibits the Protective Effect of DSS on Primary Hippocampal Neuron OGD-Induced Apoptosis

**Figure 6:**
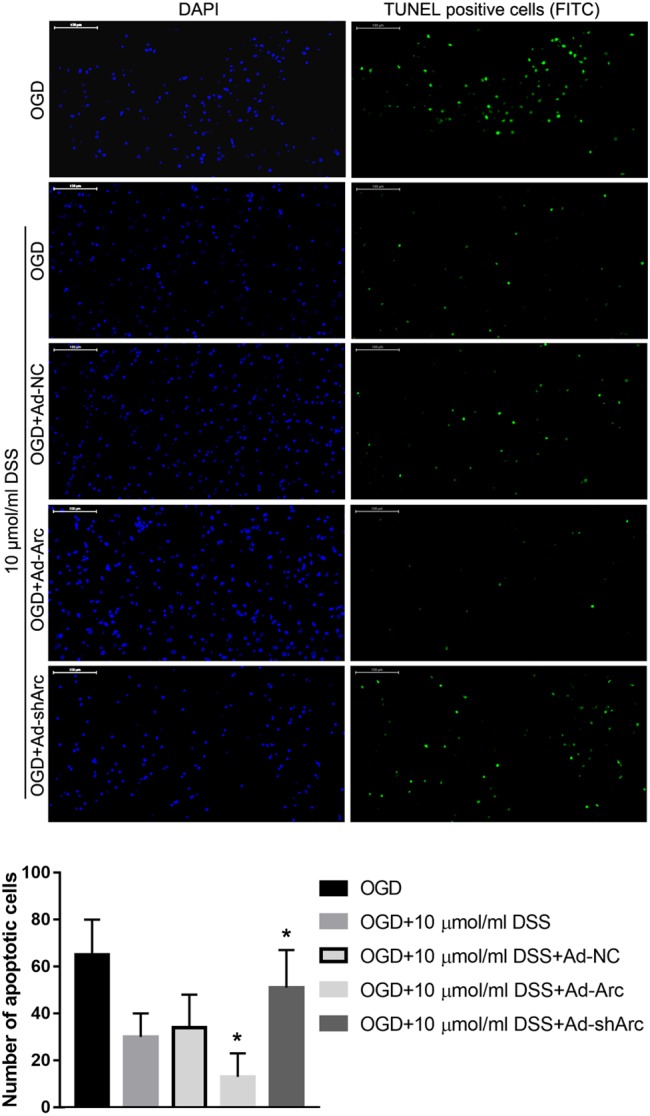
Arc overexpression enhances and Arc knockdown inhibits the beneficial effect of DSS on hippocampal neuron OGD-induced apoptosis. DAPI staining is indicated by blue nuclei. Green indicates TUNEL positive cells (apoptotic cells) stained with FITC. The bar graph represents the quantification of apoptotic cells per group. Bars represent mean ± SD for sample replications (*n* = 10). ^∗^*p* < 0.05, when compared to the OGD+10 μmol/ml DSS+Ad-NC group.

The role of Arc overexpression and silencing on the DSS-mediated neuroprotection in the *in vitro* OGD neuronal model was examined by TUNEL. The results indicate that the number of apoptotic hippocampal neurons in the OGD+10 μmol/ml DSS+Ad-Arc group was smaller than that in the OGD+10 μmol/ml DSS+Ad-NC group. Moreover, the number of apoptotic hippocampal neurons in the OGD+10 μmol/ml DSS+Ad-shArc group was higher than that in the OGD+10 μmol/ml DSS+Ad-NC group (Figure 6). These results indicate that Arc overexpression enhances and Arc knockdown inhibits the neuroprotective effect of DSS on primary hippocampal neurons following OGD neurotoxicity.

### DSS Restores the OGD-Mediated Decrease in PKA, ERK1/2, and CREB Phosphorylation in Primary Hippocampal Neurons

**Figure 7:**
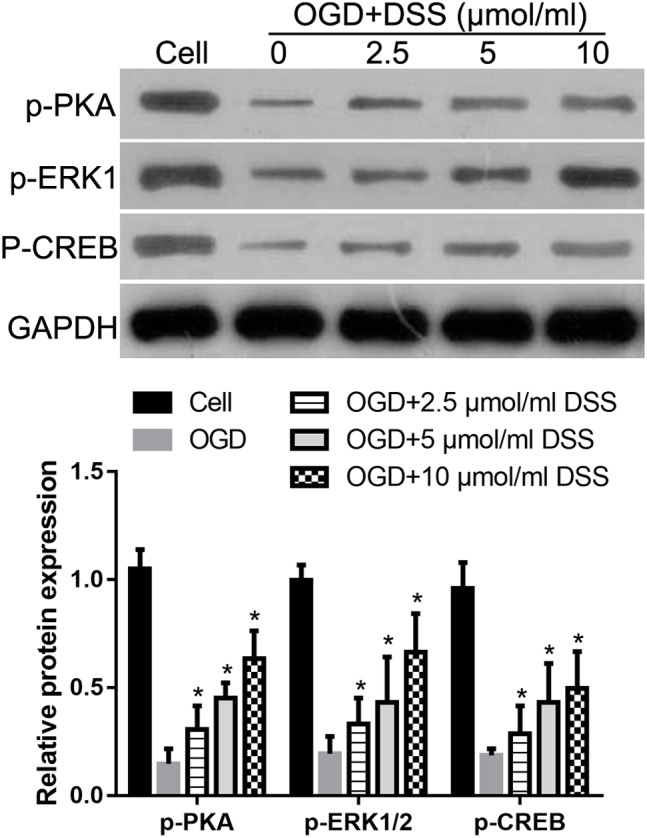
3′-Daidzein sulfonate sodium increases the phosphorylation of PKA, ERK1/2 and CREB in a primary hippocampal OGD neuronal model. Initial western blot analysis and the quantification of the relative expression of p-PKA, p-ERK1/2, and p-CREB normalized to GAPDH, is shown here. Bars represent mean ± SD for samples replications (*n* = 3). ^∗^*p* < 0.05, DSS-treated group vs. OGD group.

Our previous study indicated that DSS restores the phosphorylation levels of PKA, ERK1/2, and CREB in the hippocampus, following their 2VO-mediated decrease (Li et al., 2018b). To further confirm the effect of DSS on PKA, ERK1/2 and CREB phosphorylation in a CCH model, we examined the levels of p-PKA, p-ERK1/2, and p-CREB after treatment with different DSS concentrations in primary OGD-treated hippocampal neurons. As expected, western blotting (Figure 7) revealed that the levels of p-PKA, p-ERK1/2, and p-CREB in our *in vitro* OGD group decreased, as compared to those in the cell group. After a 2.5, 5, or 10 μmol/ml DSS treatment, p-PKA, p-ERK1/2, and p-CREB gradually increased in the primary hippocampal neurons. These results indicate that DSS can activate both PKA/CREB and ERK/CREB signaling pathways in primary OGD-treated hippocampal neurons.

### PKA or ERK1/2 Inhibition Abolishes the *in vitro* DSS-Mediated Neuroprotection and Arc Upregulation

**Figure 8:**
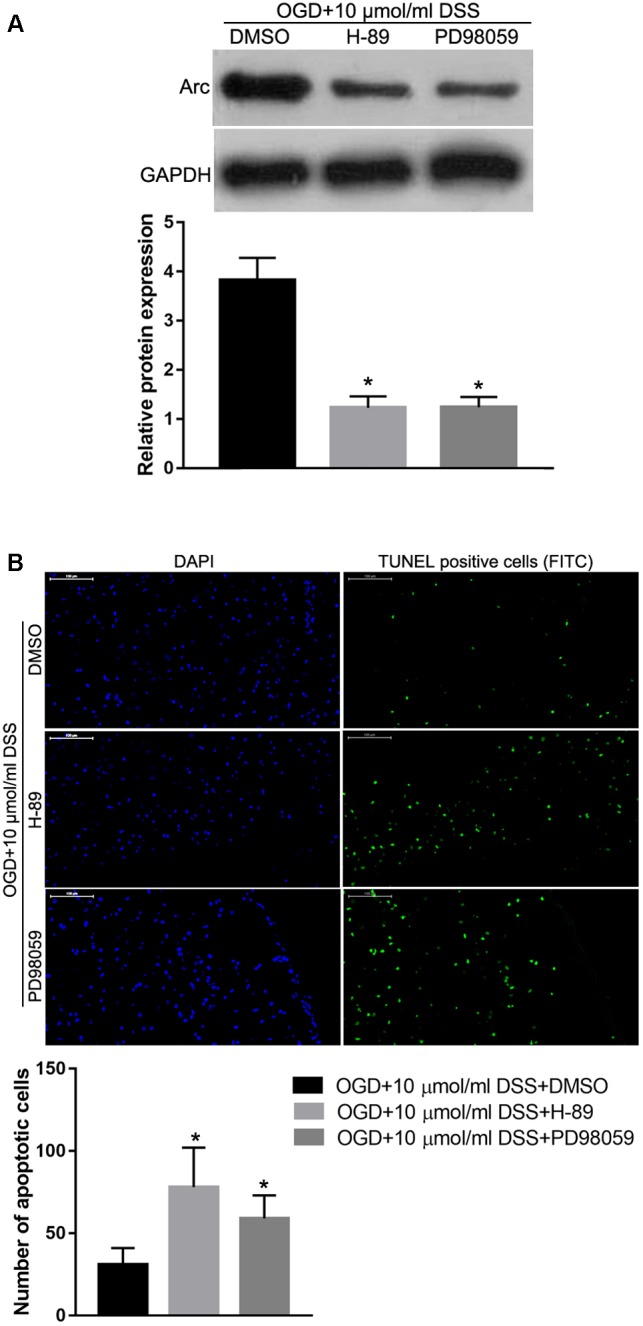
Protein kinase alpha or ERK1/2 inhibition abolishes the DSS-induced Arc upregulation and hippocampal neuroprotection in the OGD *in vitro* model. **(A)** Arc expression levels detected by Western blot. The top bar graph represents the Western blot quantification of the Arc relative expression, as normalized to GAPDH. **(B)** Results of the TUNEL assay. Blue indicates DAPI-stained nuclei. Green indicates TUNEL positive cells (apoptotic cells) stained with FITC. The bottom bar graph represents the quantification of the apoptotic neuronal cells per group. Bars represent mean ± SD for sample replications (*n* = 3). ^∗^*p* < 0.05, when compared to the OGD+10 μmol/ml DSS+DMSO group.

To identify the role of the PKA/CREB or ERK/CREB signaling pathway in regulating the effect of DSS on OGD-induced toxicity and Arc expression, the PKA inhibitor, H-89 or the ERK1/2 inhibitor, PD98059 were used to block the respective DSS-activated signaling pathways. Western blot analysis revealed that both H-89 and PD98059 significantly decreased Arc protein expression in DSS-treated OGD hippocampal neurons (Figure 8A). In addition, both H-89 and PD98059 significantly increased the number of primary DSS-treated apoptotic hippocampal neurons in our *in vitro* OGD model (Figure 8B).

## Discussion

3′-Daidzein sulfonate sodium protected against memory impairment and hippocampal damage caused by 2VO (Li et al., 2018b), indicating that DSS may be a potential drug target for the treatment of neuronal damage and cognitive impairment caused by CCH. Thus, investigating the underlying mechanisms of this DSS-mediated neuroprotection will aid in the confirmation of its clinical significance for the treatment of CCH-related diseases.

Firstly, we observed that Arc protein expression was decreased in the hippocampi of the 2VO rat model *in vivo*, as well as in hippocampal OGD-treated neurons *in vitro*. This decrease was inhibited by DSS treatment. It has been reported that Arc expression levels can be decreased by anesthetics, anesthesia-induced hypothermia, and sleep loss, which can affect many cognition aspects (Whittington et al., 2013; Delorme et al., 2018). Since CCH leads to cognitive impairment, it is easy to assume that CCH might decrease Arc expression. Even so, our study is the first report to provide evidence of this effect. Arc is critical for homeostatic synaptic scaling, long-term potentiation and depression of synaptic transmission, as well as adaptive functions, such as long-term memory formation (Nikolaienko et al., 2018). Thus, we hypothesized that Arc might play a role in the DSS-mediated protective effect against 2VO-induced memory impairment. We observed that Arc overexpression promotes DSS-mediated neuroprotection in the 2VO model and, *vice versa*, this protective effect is inhibited by Arc silencing. All of our results suggest that DSS protects against cognitive impairment caused by CCH via Arc upregulation.

Moreover, we showed that Arc overexpression alone was able to reduce neuronal apoptosis and increase neuronal densities more than DSS treatment alone, both in *in vivo* and *in vitro* hippocampal models. However, the protective effect of DSS was abolished by Arc silencing. Thus, these results suggest that DSS protects against hippocampal damage caused by CCH via the upregulation of Arc. Interestingly, our results indicate a new functional role of Arc in the regulation of neuronal apoptosis. This is a novel discovery and thus, its underlying mechanism and clinical relevance require further investigation.

Our previous study indicated that DSS restores the phosphorylation of PKA, ERK1/2, and CREB following their 2VO-induced hippocampal downregulation. CREB is a transcription factor that mediates cellular responses in response to a variety of physiological signals in the nervous system, such as neurotransmitters and synaptic activity (Ginty et al., 1994; Deisseroth et al., 1996). When CREB is activated, it binds to the CREB binding-protein and subsequently activates various target genes (Mayr and Montminy, 2001). CREB is a member of the synaptic activity-responsive element (SARE) in the Arc promoter region that enables synapse-to-nucleus signaling in activated neurons (Seibenhener and Wooten, 2012). CREB activation can be regulated by PKA and ERK1/2 (Barlow et al., 2008; Malhotra et al., 2015). We sought to examine whether DSS regulates Arc expression through PKA/CREB and/or ERK/CREB signaling. For this reason, either the PKA inhibitor, H-89, or the ERK1/2 inhibitor, PD98059, were used to block the respective signaling pathway, following activation by DSS in a hippocampal *in vitro* OGD neuronal model. Our results suggested that PKA or ERK1/2 inhibition abolishes the DSS-mediated Arc upregulation observed in the DSS-treated OGD model. This indicates that DSS regulates Arc expression through both the PKA/CREB and ERK/CREB signaling pathways. In addition, we showed that PKA or ERK1/2 inhibition also abolished the neuroprotective DSS effect exerted on primary OGD hippocampal neurons. These results indicate that DSS protects against CCH-induced neuronal damage by upregulating Arc expression through both the PKA/CREB and ERK/CREB signaling pathways. However, this finding needs further *in vivo* studies to confirm its validity.

However, the underlying mechanisms of DSS-mediated neuroprotection are complex, so transcriptomics and proteomics may be a good approach to identify other new regulating mechanisms. This is the first limitation of our study. In addition, the second limitation is that all experiments were paired with DSS treatment. Further study is needed to investigate the effect of Arc overexpression or knockdown alone on memory impairment and hippocampal damage caused by 2VO. Moreover, although the selective inhibition of H-89 on PKA is stronger, H-89 also inhibits a number of other protein kinases. Therefore, a knockdown of PKA should be carried out *in vivo* and *in vitro* to further confirm the relationship between Arc and PKA.

In conclusion, DSS protects against cognitive impairment and hippocampal damage caused by CCH via Arc upregulation, which may be induced by both the PKA/CREB and ERK/CREB signaling pathways. Our study further confirms the therapeutic potential of DSS for CCH-related disorders. Nevertheless, further studies are needed to confirm the clinical relevance of this DSS-mediated neuroprotection.

## Author Contributions

HX designed the experiments. QZ, ZH, and JDZ performed the experiments, analyzed the data, and wrote the manuscript. RL, XL, and JZ analyzed the data.

## Conflict of Interest Statement

The authors declare that the research was conducted in the absence of any commercial or financial relationships that could be construed as a potential conflict of interest.
